# Bacterial contamination of frequently touched objects in a tertiary care hospital of Pokhara, Nepal: how safe are our hands?

**DOI:** 10.1186/s13756-018-0385-2

**Published:** 2018-08-06

**Authors:** Dharm Raj Bhatta, Deependra Hamal, Rajani Shrestha, Supram Hosuru Subramanya, Nisha Baral, Rajesh Kumar Singh, Niranjan Nayak, Shishir Gokhale

**Affiliations:** Department of Microbiology, Manipal College of Medical Sciences, Pokhara, Nepal

**Keywords:** Hospital surfaces, Bacterial contamination, *Staphylococcus aureus*, MRSA, Drug resistance, Cleaning, Disinfection, Biofilm

## Abstract

**Background:**

Objects frequently touched by patients and healthcare workers in hospitals harbor potential pathogens and may act as source of infectious agents. This study aimed to determine the bacterial contamination of common hospital objects frequently touched by patients, visitors and healthcare workers.

**Methods:**

A total of 232 samples were collected from various sites like surface of biometric attendance devices, elevator buttons, door handles, staircase railings, telephone sets and water taps. Isolation, identification and antibiotic susceptibility testing of the isolates was performed by standard microbiological techniques. Biofilm forming ability of the *S. aureus* isolates was tested by a microtitre plate method.

**Results:**

A total of 232 samples were collected and 219 bacterial isolates were recovered from 181 samples. *Staphylococcus aureus* was the most common bacterial isolate (44/219). Majority of *S. aureus* isolates were recovered from elevator buttons, biometric attendance devices and door handles. Among the *S. aureus* isolates, 36.3% (16/44) were methicillin resistant *Staphylococcus aureus* (MRSA) while remaining were methicillin sensitive *Staphylococcus aureus* (MSSA). Out of 44 *S. aureus* isolates, 12 (29.5%) were multidrug resistant and 14 (31.8%) were biofilm producers. The majority of MRSA isolates 62.5% (10/16) were biofilm producers. *Acinetobacter* was the most common Gram negative isolate followed by *E coli* and *Pseudomonas* species.

**Conclusions:**

High bacterial contamination of frequently touched objects with variety of potential pathogens and normal flora was detected. *S. aureus* was the most common bacterial isolate. Biofilm forming ability offers additional survival advantage to the organisms on these objects. Present study highlights the need of improved hand hygiene among healthcare workers and regular cleaning/disinfection of sites of frequent public contact.

## Background

Hospital environment, objects/instruments and healthcare workers are likely to get colonized with diverse group of microbial agents. The direct contact with infected and/or colonized patients as well as objects may lead to transmission, resulting into morbidity and mortality [1, 2]. In the United States, every year approximately two million patients acquire nosocomial infection and at least 90,000 of them die [3, 4]. Nosocomial infections are the fifth leading cause of death in critical-care hospitals. The prevalence of hospital acquired infections (HAI) in developing countries is neither recorded nor reported properly due to various reasons [5]. A systemic review has estimated the prevalence of HAIs to be 7.6% in high income countries and 10.1% in low and middle income countries [6].

It has been reported that, bacteria can survive for variable duration on surfaces including white coats, stethoscopes, adhesive tape, computer keyboards, elevator buttons, mobile communication devices, and ultrasound transducers [7, 8]. Risk of transmission is directly proportional to the duration of survival of the bacteria on the colonized objects. The colonization and survival depends on geographical and environmental conditions like temperature, humidity, presence of organic matter, ability to form biofilms and the prevalent infection control practices [9, 10].

Gram positive and Gram negative bacteria have been reported to survive up to months on dry inanimate surfaces in the hospitals [9]. Nosocomial bacterial pathogens like methicillin resistant *Staphylococcus aureus* (MRSA), Vancomycin resistant *Enterococci* (VRE), *Pseudomonas* spp., *Acinetobacter* spp. are more stable in the hospital environment. The well-known pathogens like *Streptococcus pneumoniae*, *Streptococcus pyogenes* and *Haemophilus influenzae* are delicate, fastidious and quickly inactivated after excretion from the patients, hence have short survival on inanimate surfaces. The organisms from objects of frequent contact in hospital are likely to get transmitted to healthcare workers, patients and visitors.

This study was planned to investigate the diversity and distribution of bacterial contamination of frequently touched surfaces shared by healthcare workers, patients and visitors. These sites were targeted because these are frequently touched but most neglected from cleaning/disinfection procedures. Limited data is available regarding bacterial colonization of frequently touched inanimate objects in hospital setting of Nepal. Identification of these sites and bacterial agents may help to reduce transmission of pathogens.

## Methods

This hospital based prospective study was performed at Manipal Teaching Hospital, Pokhara, Nepal, over a period of five months. Manipal Teaching Hospital is a 750 bedded tertiary care hospital having departments of Medicine, Surgery, Pediatrics, Obstetrics and gynaecology, Orthopedics, Otorhinolaryngology, Ophthalmology, Psychiatry, Dermatology Cardiology, Gastroenterology, Urology and Oncology providing care to variety of patients. Approval from the Institutional Ethical Committee (IRC) of Manipal College of Medical Sciences, Pokhara, Nepal, was obtained before commencement of the study. The cleaning and disinfection for floor, table tops, working benches, invasive instruments, Operation Theater (OT) and intensive care units (ICUs) were observed. The floor is wet mopped twice a day in general wards and with detergent solution in ICUs. In addition, automated mopping machine scrubs corridor floor on weekly basis. Table tops and benches are wiped twice a day with 70% Isopropyl alcohol swabs. Invasive instruments are disinfected with Cidex as per manufacturer’s directions. Fumigation of OT, ICUs is performed by fumigator filled with quaternary ammonium compound solution (SOT 125 TM) on weekly basis. There are no established practices of cleaning/disinfecting surface of biometric attendance devices, elevator buttons, door handles, staircase railings, water taps and telephone sets.

### Specimen collection

A total of 232 samples were collected from various surfaces like biometric finger print devices, elevator buttons, door handles, staircase railings, telephone sets and water taps. Majority of these sites are accessible and commonly used by patients, visitors and healthcare professionals. The most frequently touched surfaces samples were collected from target sites by rubbing and rotating sterile swabs moistened with normal saline. The departments were selected based on the patients load. The most occupied departments such as Medicine, Surgery, Intensive Care Units were included. Length of the swabs used for sampling was approximately 1.5 cm. The available whole surface area of the finger print (2.5 cm × 1.5 cm), elevator buttons (2 cm × 2 cm), water tap handles (3.5 cm × 1 cm) and door handles (7 cm × 1 cm) was sampled. Handset (20 cm × 3.5 cm) and number pad (7 cm × 6 cm) of telephone sets were sampled. Upper part of staircase railing was randomly sampled.

### Isolation and identification of bacterial isolates

Samples were inoculated immediately at the point of collection by rotating and rolling on the surface of 5% Sheep Blood agar and MacConkey agar plates. Inoculated plates were immediately transported to the laboratory and incubated at 37 °C. Identification of the isolates was performed by standard microbiological techniques such as colony morphology, microscopic features and standard phenotypic characters [11].

### Antibiotic susceptibility test

Antibiotic susceptibility testing of the isolates was performed on Mueller Hinton agar (HI media, Mumbai, India) by Kirby Bauer disc diffusion method [12]. Bacterial isolates resistant to at least one agent in three or more antimicrobial categories were labeled as multidrug resistant (MDR) [13]. Methicillin resistant *Staphylococcus aureus* (MRSA) were screened by cefoxitin (30 μg) disc diffusion method [12]. Extended Spectrum Beta Lactamase (ESBL) production among Gram negative bacilli was performed by standard methods [12].

### Biofilm detection

Biofilm forming ability of the *S. aureus* isolates was tested by crystal violet staining method as described by Christensen et al. [14], with minor modifications. Overnight cultures of bacteria in brain heart infusion broth with 1% glucose were diluted 1:100 in fresh broth (BHIB) and 200ul of each were added to separate wells of the microtitre plate. Ten different wells containing 200 ul of media (BHIB) for each batch of the test served as controls. Microtitre plate was then covered with aluminum foil and incubated at 37 °C. After overnight incubation, the wells were washed four times with sterile phosphate buffer saline, fixed with Boine’s fixative (saturated solution of picric acid 75 parts, formaldehyde 25 parts and glacial acetic acid 5 parts) and then stained with Hucker’s crystal violet. Excess stain was removed by decanting the plate. The plate was then left at room temperature for 15 min followed by three washing. After this, 200 μl of alcohol: acetone mixture (80:20 *w*/*v*) was added to each well. 100 μl of the mixture from each well was transferred to another microtitre plate and absorbance was recorded at 450 nm. Biofilm producers and non-biofilm producing standard strains of *S. aureus* served as positive and negative controls respectively. The cut off OD (three times the standard deviation above the mean OD) was calculated and used for differentiating biofilm producers and non-biofilm producers. The OD values higher than cut off were considered as biofilm positive.

## Results

Out of 232 samples collected from various sites, bacterial growth was observed in 181 (78%) specimens while remaining 51 (22%) did not show bacterial growth. A total of 219 bacterial isolates were cultured from 181 sites. Mixed bacterial flora was isolated from various sites. *Staphylococcus aureus* was the most common isolate cultured from 44 different sites. Details of specimen and bacterial isolates are depicted in Table 1. Majority of *S. aureus* isolates were from door handles 29.5% (13/44), elevator buttons 25% (11/44) and biometric attendance devices 18.1% (8/44). Among *S. aureus* isolates, 36.3% (16/44) were MRSA and remaining were MSSA. The antimicrobial resistance pattern of *S. aureus* isolates is shown in Table 2. All the isolates of *S. aureus* were susceptible to amikacin.

**Table 1:** Bacteria isolated from environmental surfaces

Organisms	Elevator buttons (*n* = 48)	Biometric attendance devices (*n* = 24)	Door handles (*n* = 80)	Telephone sets (*n* = 30)	Railing (*n* = 20)	Water taps (*n* = 30)
*S. aureus*	11 (22.9%)	08 (33.3%)	13 (16.2%)	06 (20%)	03 (15%)	03 (10%)
*S. epidermidis*	10 (20.8%)	06 (25%)	11 (13.7%)	04 (13.3%)	01 (5%)	02 (6.6%)
*Enterococcus* species	04 (8.3%)	05 (20.8%)	08 (10%)	06 (20%)	02 (10%)	03 (10%)
*Diphtheroids*	05 (10.4%)	04 (16.6%)	07 (8.7%)	08 (26.6%)	04 (20%)	07 (23.3%)
*Micrococcus* species	07 (14.6%)	05 (20.8%)	08 (10%)	11 (36.6%)	02 (10%)	04 (13.3%)
*Escherichia coli*	03 (6.2%)	02 (8.3%)	04 (5%)	03 (10%)	01 (5%)	02 (6.6%)
*Pseudomonas* species	02 (4.1%)	03 (12.5%)	–	–	–	02 (6.6%)
*Acinetobacter* species	02 (4.1%)	03 (12.5%)	04 (5%)	05 (16.6%)	01 (5%)	04 (13.3%)
Total	44	36	55	43	14	27

**Table 2:** Antibiotic resistance pattern of *Staphylococcus aureus* (MRSA and MSSA) isolates

Antibiotic	*S. aureus* isolates (*n* = 44) Frequency (%)	MRSA isolates (*n* = 16) Frequency (%)	MSSA isolates (*n* = 28) Frequency (%)
penicillin	40 (90.9)	16 (100)	24 (85.7)
erythromycin	25 (56.8)	16 (100)	09 (32.1)
ciprofloxacin	7 (15.9)	06 (37.5)	1 (3.5)
gentamicin	02 (4.5)	02 (12.5)	00
clindamycin	07 (15.9)	05 (31.2)	02 (7.1)
ceftriaxone	11 (25)	10 (62.5)	01 (3.5)
cotrimoxazole	11 (25)	08 (50)	03 (10.7)
amikacin	00	00	00

MRSA: Methicillin resistant *Staphylococcus aureus*, MSSA: Methicillin sensitive *Staphylococcus aureus*

Among Gram negative bacteria, *Acinetobacter* species, *E coli* and *Pseudomonas* species were isolated. Majority of *Acinetobacter* species 52.6% (10/19) and *E coli* 46.6% (7/15) were ESBL producers. All the isolates of *Acinetobacter* species, *E coli* and *Pseudomonas* species were susceptible to amikacin and imipenem. The antimicrobial resistance pattern of Gram negative isolates is depicted in Table 3.Out of 44 *S. aureus* isolates, 12 (29.5%) were multidrug resistant and 14 (31.8%) were biofilm producers. The majority of MRSA isolates 62.5% (10/16) were biofilm producers (Table 4).

**Table 3:** Antibiotic resistance pattern of Gram negative isolates

Antibiotic	*Acinetobacter* species *n* = 19 (%)	*Escherichia coli n* = 15 (%)	*Pseudomonas* species *n* = 07 (%)
ciprofloxacin	11 (57.9)	10 (66.6)	04 (57.1)
ceftazidime	12 (63.1)	09 (60)	05 (71.4)
cotrimoxazole	09 (47.3)	07 (46.6)	04 (57.1)
cefotaxime	12 (63.1)	09 (60)	05 (71.4)
gentamicin	01 (5.2)	02 (13.3)	02 (28.5)
imipenem	0	0	0
amikacin	0	0	0

**Table 4:** Biofilm production among MRSA and MSSA isolates

Organisms	Biofilm production		Total
	Positive	Negative	
MRSA	10 (62.5%)	06 (37.5%)	16
MSSA	04 (14.3%)	24 (85.7%)	28
Total	14 (31.8%)	30 (68.2%)	44

MRSA: Methicillin resistant *Staphylococcus aureus*, MSSA: Methicillin sensitive *Staphylococcus aureus*

## Discussion

Microbial population constantly inhabit community and hospital environment. The hospital surfaces are often contaminated with flora excreted by patients, visitors and healthcare workers. The contaminated environmental surfaces are potential reservoirs for spread of microbial agents in hospital as well as community. Persistence of pathogens in hospital environment increases the risk of infection among susceptible host [15]. Microbial population and colonization rate vary with different hospitals of different countries. Study of bacteriological profile of common sites of hand contact would help to locate the possible reservoirs of bacteria and to apply suitable disinfection techniques.

The bacteriological profile of frequently used objects (Non medical devices) yielded wide variety of organisms ranging from normal flora to potential pathogens like *S. aureus*, *Acinetobacter* species, *E coli* and *Pseudomonas* species. The biometric attendance devices are widely used in hospital as well as community. Microbial colonization of such devices may spread the potential pathogens among unsuspecting users. In our study, 33.3% (8/24) specimens collected from biometric attendance devices showed growth of *S. aureus* of which 25% (2/8) were MRSA. *Escherichia coli* is commonly associated with community infections, isolation of ESBL producing *E coli* from these sites indicates possible role in serious nosocomial infections. *Acinetobacter* species and *Pseudomonas* species are well known nosocomial pathogens and their presence on biometric devices can lead to spread in the hospital and community. Similar studies have shown the presence of *Acinetobacter* species, *Pseudomonas* species and *E coli* on other inanimate objects of hospital [16, 17]. Manipal Teaching hospital has more than six hundred healthcare workers who mark attendance twice a day with these devices. Colonization of such devices by potential pathogens like MRSA and ESBL producing Gram negative bacteria indicates the possible spread of these pathogens among the hospital as well as in community population. Most of the healthcare workers before starting their duty and at the end of duty hours mark attendance with biometric devices. At the end of duty hours, healthcare workers wash their hands in their respective departments and then mark attendance on biometric devices. Their fingers may get contaminated with pathogens persisting on the devices. Healthcare workers rarely wash hands after use of biometric attendance devices. This may lead to dissemination of pathogens from hospital into community. Nancy S et al also reported 33.3% *S. aureus* isolation and 70% MRSA prevalence on biometric device surfaces [18].

Hospital elevator buttons are another frequently touched objects by healthcare workers, patients and visitors. Out of 48 specimens collected from elevator buttons, 22.9% (11/48) showed growth of *S. aureus* with 36.3% (4/11) MRSA. Sayeed et al reported 75% contamination of elevator buttons with *S. aureus* which is higher than our findings [19]. Other potential pathogens were *Pseudomonas* species, *Acinetobacter* species and *E coli*. Kandel et al, reported isolation of *Staphylococcus* species, *Pseudomonas* species, coliform bacteria from elevator buttons [20]. Healthcare workers including doctors and nurses frequently use elevators. This increases the risk of transmission of these potential pathogens to the patients. Similarly, finger contamination of medical students, visitors and staff by elevator buttons may spread these pathogens in the community as well. Elevator buttons are one of the neglected sites in a hospital, often not cleaned or disinfected and can become potential site for bacterial colonization.

Door handle contamination by potential pathogens has been recorded from the medical ward, surgical ward, ICU and post-operative ward. In a study by Odigie et al., *S. aureus*, *Pseudomonas* species and *E coli* were reported as common isolates from door handles [21]. In a recent study, *E coli* was reported second most common bacterial isolate from door handles [22]. Isolation of *E coli* from door handles of pediatric units could be threat for serious infections among neonates. In a similar study by Oie S et al, door handle contamination by *S. aureus* in a University hospital in Japan was 27% which is comparable with our study (16.2%) [23]. Saba et al, reported *S. aureus* colonization rate of 39% (47/120) from door handles, staircase railings and other point of contact in Teaching Hospitals [24]. The MRSA colonization on door handles in our study was 30.7% (4/13) which is higher as compared to above study (17%). The risk of transmission of pathogens is more as nursing staff, clinicians and visitors frequently touch door handles during visit to wards.

Hospital telephone sets can be important source of nosocomial pathogens in various units. Contamination is likely to occur due to regular hand and ear contact of healthcare workers with almost no attempt at cleaning/disinfection. The contamination of telephone sets by potential pathogens was observed from Surgery, ICU, Pediatric, Gyanecology and Medicine wards. Culture of telephone sets yielded large number of *Acinetobacter* species. Isolation of multidrug resistant *Acinetobacter* species and *E coli* from ICU is alarming. *S. aureus* colonization rate of telephone sets was 20% (6/30) with 33.3% (2/6) MRSA. In a similar study by Zubair et al., lower, 9% (4/44) *S. aureus* contamination was reported [25]. Bacterial contamination of other frequently touched sites like staircase railings pose the risk of transmission especially among the children who fondle railings during hospital visit. Similarly, contamination of water taps (wash room and drinking water) by nosocomial pathogens may lead to contamination of drinking water resulting into gastrointestinal disorders. Presence of potential pathogens on hand operated water taps of washroom increases the possibility of recontamination of hands negating the benefits of hand washing. Above mentioned sites were selected for this study due to frequent and unavoidable contact with these surfaces. These sites are very infrequently cleaned or decontaminated. In most of the teaching hospitals, routine cleaning involves hospital floor, working bench, table tops, nursing stations, dressing trolley etc. However, there is almost no practice of cleaning/disinfecting sites highlighted in this study. High rate of bacterial contamination in the above mentioned sites reflects poor hand hygiene among the healthcare workers and visitors as transmission occurs mainly through contaminated fingers.

*Staphylococcus aureus* is well known nosocomial pathogen with its ability to survive in hospital environment for several days [26]. The ability of *S. aureus* and MRSA to form biofilm on inanimate objects prolongs their survival and spread. Ankit et al. from Nepal, reported 65.7% clinical isolates of MRSA from pus/wound swabs as biofilm producers, however data related to environmental samples is not available [27]. Majority of MRSA isolates 62.5% (10/16) in our study were biofilm producers which is comparable with above mentioned report. This is alarming as MRSA isolates embedded in biofilm can survive long duration and can become potential source of MRSA associated nosocomial and community infections. Such infections are difficult to treat due to in vivo biofilm formation. Identification of the more frequently contaminated sites and the most commonly identified potential pathogen is important for infection control practices and promotion of new interventions [28].

Study of bacterial contamination of multiple sites of frequent hand contact in a hospital is the strength of our study. Biofilm formation by the most common potential pathogen *S. aureus* is important determinant for their survival and transmission.

This study has some limitations. The molecular characterization of the potential pathogens was not performed. We could not prove the association of pathogens isolated from objects and prevalent nosocomial infections. Biofilm property was studied only for *S. aureus* isolates. The study was conducted in one of the tertiary care hospital and results of the study may not be generalized.

## Conclusion

This is probably the first study in Nepal assessing bacterial contamination of objects most frequently touched but neglected for cleaning/disinfection. Isolation of potential pathogens like MRSA, *Acinetobacter* species, *E coli* from common point of contact poses threat of transmission among hospital and community population. Findings of this study are important to emphasize hand hygiene and decontamination of these sites on a regular basis. Availability and use of hand washing/hand sanitizer after contact with biometric system, elevator button or door handles may reduce the possibility of transmission of potential pathogens. Decontamination of the sites with alcohol based disinfectants would reduce the microbial flora from these sites. This process would be convenient as most of these sites have small surface area which can be covered in short period of time. Provision of non-hand touch techniques for doors, taps/faucets could be another way of reducing the transmission.
